# GPR162 is a beta cell CART receptor

**DOI:** 10.1016/j.isci.2023.108416

**Published:** 2023-11-10

**Authors:** Andreas Lindqvist, Mia Abels, Liliya Shcherbina, Mtakai Ngara, Dmytro Kryvokhyzha, Sabrina Chriett, Matteo Riva, Abul Fajul, Mohammad Barghouth, Cheng Luan, Lena Eliasson, Olav Larsen, Mette M. Rosenkilde, Enming Zhang, Erik Renström, Nils Wierup

**Affiliations:** 1 Neuroendocrine Cell Biology, Lund University Diabetes Centre, Department for Experimental Medical Science, Lund University, CRC, Malmö, Sweden; 2 Diabetic Complications, Lund University Diabetes Centre, Department of Clinical Sciences Malmö, Lund University, CRC, Malmö, Sweden; 3 Islet Pathophysiology, Lund University Diabetes Centre, Department of Clinical Sciences Malmö, Lund University, CRC, Malmö, Sweden; 4 Islet Cell Exocytosis, Lund University Diabetes Centre, Department of Clinical Sciences Malmö, Lund University, CRC, Malmö, Sweden; 5 Department of Biomedical Sciences, University of Copenhagen, Copenhagen, Denmark

**Keywords:** Natural sciences, Biological sciences, Physiology, Endocrinology

## Abstract

Cocaine and amphetamine-regulated transcript (CART) is expressed in pancreatic islet cells and neuronal elements. We have previously established insulinotropic actions of CART in human and rodent islets. The receptor for CART in the pancreatic beta cells is unidentified. We used RNA sequencing of *Cartpt* knockdown (KD) INS-1 832/13 cells and identified GPR162 as the most *Cartpt*-regulated receptor. We therefore tested if GPR162 mediates the effects of CART in beta cells. Binding of CART to GPR162 was established using proximity ligation assay, radioactive binding, and co-immunoprecipitation, and KD of *Gpr162* mRNA caused reduced binding. *Gpr162* KD cells had blunted CARTp-induced exocytosis, and reduced CARTp-induced insulin secretion. Furthermore, we identified a hitherto undescribed GPR162-dependent role of CART as a regulator of cytoskeletal arrangement. Thus, our findings provide mechanistic insight into the effect of CART on insulin secretion and show that GPR162 is the CART receptor in beta cells.

## Highlights

• The orphan receptor GPR162 is affected by *Cartpt* knockdown in beta cells
• CART and GPR162 are shown to interact using a number of binding assays
• CART-induced effects are reduced/abolished after knockdown of *Gpr162* in beta cells
• *Gpr162* knockdown alters CART-driven processes, including cytoskeletal architecture

## Graphical abstract

**Figure:**
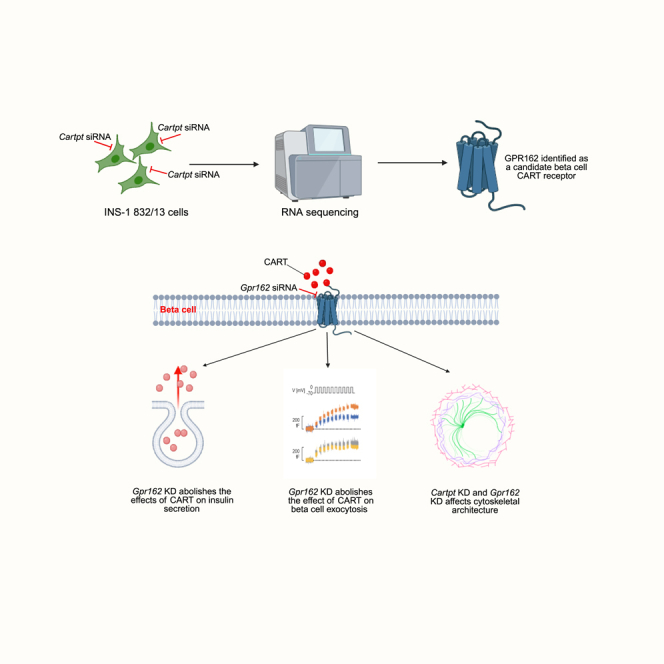

## Introduction

In 1995, cocaine- and amphetamine-regulated transcript (CART) was identified as a transcript being upregulated in rat striatum in response to psychostimulant drugs.^1^ However, much earlier, a fragment of the CART peptide was identified in extracts of sheep hypothalamus.^2^ CART was later found to be expressed also in pancreatic islet cells and in nerve fibers innervating the islets.^3^^,^^4^^,^^5^ Initially, CART was found to have anorexigenic effects.^6^ Since then, multiple biological effects of CART have been established.^4^^,^^7^ Among them is the effect of CART on islet hormone secretion from the pancreatic islets. We have established that CART increases insulin secretion, but reduces glucagon secretion, both *in vivo* and *in vitro/ex vivo* in rodent and human islets.^8^ However, the understanding of the molecular basis for the effects has been hampered by the lack of an identified CART receptor. Vicentic et al. suggested in 2005 that the CART receptor is a G protein-coupled receptor as they found radiolabeled CART to be displaced by CARTp55-102, but not by non-active forms of CARTp, and that binding was reduced in the presence of a GTP analog in AtT20 cells.^9^ Furthermore, by using radiolabeled CART peptide, Kuhar and colleagues were able to detect high-affinity-specific binding in PC12 cells.^10^ This binding was found to increase upon differentiation of the cells and was inhibited by actinomycin D or cycloheximide treatment, suggesting that the observed binding of CART is dependent on RNA and protein synthesis. It was also reported that PACAP6-38 acts as a CART receptor antagonist and that CARTp-mediated phosphorylation of ERK is inhibited by pertussis toxin.^10^

When studying neuropathic pain, Yosten et al.^11^ found GPR160 to be necessary for CARTp-mediated cell activation in cell culture. RNA interference of *Gpr160* prevented CART from activating c-FOS expression and phosphorylation of ERK *in vitro* in KATO III cells and PC-12 cells, respectively.^11^ Also, intrathecal injections of CART induced phosphorylation of ERK and CREB in the dorsal horn of the spinal cord.^11^ Furthermore, intracerebroventricular administration of a GPR160 antibody to the 4^th^ ventricle increased food intake in rats.^12^ However, whether GPR160 is a receptor for CART in the beta cell was not investigated by Yosten et al.^11^ or Haddock et al.^12^ and subsequently the identity of the beta cell CART receptor remains to be resolved. It should be noted that the data by Yosten et al. and Haddock et al. were recently challenged.^13^

In the present study, we set out to identify a potential CART receptor in beta cells, using a transcriptomics approach, binding studies, as well as secretion studies, gene expression, and studies showing lack of CART effects after small interfering RNA (siRNA) knockdown. The data presented here point to GPR162 being the beta cell receptor for CART.

## Results

### RNA sequencing of *Cartpt* KD INS-1 832/13 cells reveals GPR162 to be a candidate receptor for CART

**Figure 1:**
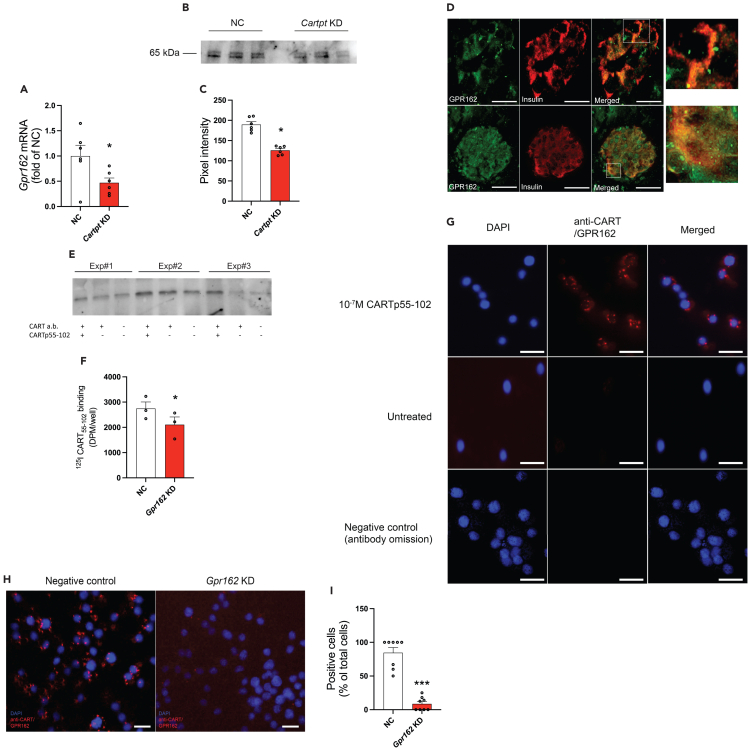
GPR162 is expressed in pancreatic beta cells and interacts with CART in INS-1 832/13 cells (A) *Cartpt* KD results in reduced *Gpr162* mRNA expression (n = 6 biological replicates). NC, negative control; KD, knockdown. Data are presented as mean ± SEM. ∗p < 0.05. (B) GPR162 protein expression (representative image). NC, negative control; KD, knockdown. (C) Quantification of Western blot in B (n = 6 biological replicates). NC, negative control; KD, knockdown. Data are presented as mean ± SEM. ∗p < 0.05. (D) Double immunostaining for GPR162 and insulin in human (upper row of panels) and mouse (lower row of panels) pancreatic sections (n = 4 samples for both species). Scale bar is 50 μm. (E) Co-immunoprecipitation of INS-1 832/13 cells incubated with 10^−7^M CARTp55-102 (n = 3 biological replicates; +/+ indicates initial precipitation of samples using CART antibody and incubation with CART peptide; +/− indicates precipitation with CART antibody but without CART peptide; −/− indicate absence of both initial precipitation with CART antibody and incubation with CART peptide). a.b., antibody. (F) ^125^I-CARTp55-102 binds to a lower extent to *Gpr162* KD INS-1 832/13 cells (n = 3 biological replicates). NC, negative control; KD, knockdown. Data are presented as mean ± SEM. ∗p < 0.05. (G) Proximity ligation assay visualizing the interaction between CART and GPR162 (n = 8 biological replicates). Scale bar is 20 μm. Untreated refers to cells not incubated with CARTp. (H) Proximity ligation assay in *Gpr162* KD INS-1 832/13 cells. NC, negative control; KD, knockdown. Scale bar is 20 μm. (I) Quantification of H (n = 8 biological replicates). NC, negative control; KD, knockdown. Data are presented as mean ± SEM. ∗∗∗p < 0.005. Data were analyzed using Student's t test (Figures 1A, 1C, 1F, and 1I).

In an attempt to understand the mechanisms behind the insulinotropic effects of CART in pancreatic beta cells, RNA sequencing of INS-1 832/13 cells, subjected to siRNA-mediated knockdown (KD) of *Cartpt*, was performed. In total, 5027 genes were found to be differentially expressed (2532 genes were upregulated and 2495 genes were downregulated) (Tables S1 and S2). Reasoning that KD of a ligand could affect the expression of its cognate receptor, we primarily focused on orphan receptors that were affected by *Cartpt* KD. This way we identified *Gpr162* as the orphan receptor to be most significantly affected by *Cartpt* KD and which had the highest fold change in its expression (p = 6.9∗10^−90^, log fold-change: −1.4). Reduced *Gpr162 mRNA* expression after *Cartpt* KD was confirmed with qPCR in INS-1 832/13 cells (Figure 1A) and by Western blotting (Figure 1B). Using a targeted approach, we previously established that *Cartpt* KD affects a wide range of genes crucial for beta cell function and insulin secretion and production.^14^ To assess the overall impact of *Cartpt* KD on cellular function, systematic Gene Ontology (GO) term analysis was performed. This revealed that, among others, processes related to cytoskeletal rearrangement were affected in *Cartpt* KD cells. Genes including *Rhob*, *Sec23a*, *Rab3a*, *Rab7a*, *Iqgap1*, *Rabgef1*, and *Arhgap27* were upregulated by *Cartpt* KD, while *Arhgap36*, *Rhoq*, *Iqgap2*, *Rhobtb3*, *Rab15*, and *Rab12* were downregulated by *Cartpt* KD. Other processes affected by *Cartpt* KD included sulfur compound biosynthetic process (GO:0044272, upregulated), glycerolipid biosynthesis (GO:0045017, upregulated), phospholipid biosynthetic process (GO: 0008654, upregulated), glycerophospholipid metabolic process (GO:0006650, upregulated), synapse organization (GO:0050808, downregulated), cell junction organization (GO:0034330, downregulated), cell morphogenesis involved in differentiation (GO: 0000904, downregulated), and cell morphogenesis (GO:0000902, downregulated) (Table S3).

### Binding assays confirm the interaction between CART and GPR162

Based on the findings from the RNA sequencing of *Cartpt* KD INS-1 832/13 cells, we set out to investigate the possibility that GPR162 acts as a CART receptor in beta cells. Since CART is known to affect human and murine secretion of islet hormones, we first confirmed GPR162 mRNA expression (Figure 1A) and protein expression (Figures 1B and 1C) in INS-1 832/13 cells. We also show that *Cartpt* KD in INS-1 832/13 cells affects *Gpr162* mRNA expression and GPR162 protein expression (Figures 1A, 1B, and 1C, respectively). Furthermore, we confirmed GPR162 expression in mouse and human islets (Figure 1D). In line with CART affecting both insulin and glucagon secretion,^8^^,^^15^ GPR162 was localized to beta cells (Figure 1D) and alpha cells (Figure S1D). Moderate GPR162 immunoreactivity was evident in the cytoplasm and more intense immunoreactivity was seen in hitherto unidentified cellular structures. In human islets 82% ± 13% of the beta cells were GPR162 immunoreactive and the corresponding number was 74% ± 20% for mouse beta cells. A similar expression pattern in beta cells was seen using two different GPR162 antibodies (Figure S1A), and pre-absorption experiments further added to the specificity of the antibody (Figure S1B). Furthermore, *Gpr162* KD (using two different siRNAs) caused a robust reduction in GPR162 immunoreactivity in INS-1 832/13 (Figure S1C). Having established the presence of GPR162 in alpha and beta cells, we then employed a multi-technique strategy to test whether CART binds to GPR162 using INS-1 832/13 cells as a model for beta cells. Firstly, we performed co-immunoprecipitation which revealed a stronger binding for GPR162 in the CARTp-treated cells than the binding observed in control cells (Figure 1E). Furthermore, we showed that radioactive ^125^I-CARTp55-102 binds to INS-1 832/13 cells, and that the degree of binding was lower in cells treated with *Gpr162* siRNA (Figure 1F). Reduced GPR162 protein level after *Gpr162* KD was verified by western blot (Figure S1D). Moreover, proximity ligation assay (PLA) confirmed an interaction between GPR162 and CART (Figure 1G). Notably, cells treated with *Gpr162* siRNA displayed 90% lower CART-GPR162 interaction, compared with control cells (Figures 1H and 1I). The specificity of the PLA is shown in Figure S2; in a control experiment, no interaction was found between CART and the GIP receptor, a receptor with a known ligand.

While these studies were in progress, GPR160 was presented as a CART receptor in KATOIII and PC-12 cells.^11^ Although GPR160 was not among the differentially expressed genes after *Cartpt* KD (Tables S1A and S1B), we examined possible expression of GPR160 in islets and interaction between GPR160 and CART in beta cells. We failed to detect GPR160 immunoreactive cells in human islets or GPR160-CART interaction through PLA in INS-1 832/13 cells (Figures S4 and S5, respectively). The specificity of the GPR160 antibody is indicated in Figures S3C–S3F which shows robust expression of GPR160 in tissues previously reported to express GPR160.

### Knockdown of *Gpr162* reduces CART-induced exocytosis

**Figure 2:**
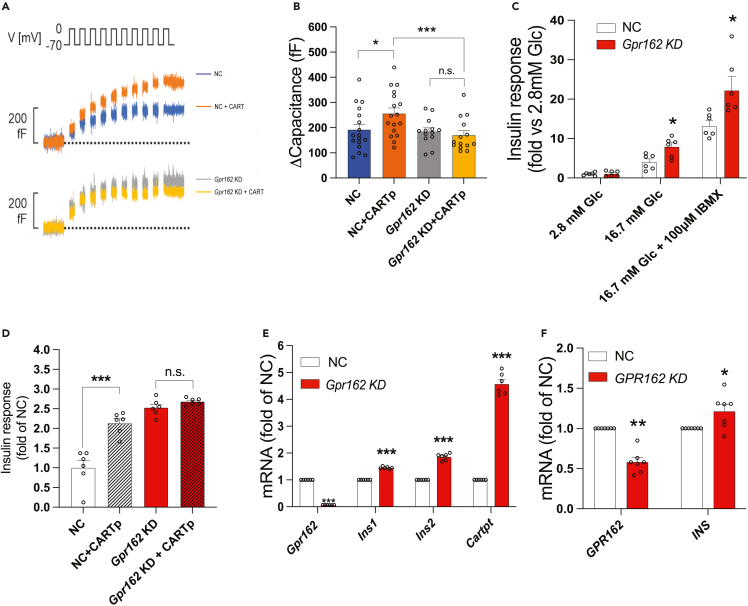
Knockdown of *Gpr162* mRNA in INS-1 832/13 cells alters CART-induced effects on beta cell function (A) Whole-cell patch-clamp experiments in INS-1 832/13 cells (n = 17 cells for NC, n = 17 cells for NC + CARTp, n = 13 cells for *Gpr162* KD, and n = 14 cells for *Gpr162* KD + CARTp). (B) Quantification of (A). NC, negative control; KD, knockdown. Data are presented as mean ± SEM. ∗p < 0.05, ∗∗∗p < 0.005. (C) Glucose-stimulated insulin secretion in *Gpr162* KD INS-1 832/13 cells (n = 6 biological replicates). NC, negative control; KD, knockdown. Data are presented as mean ± SEM. ∗p < 005. ∗ above column indicate comparisons with NC within the same glucose condition. (D) 24-h incubation with CARTp55-102 in *Gpr162* KD INS-1 832/13 cells (n = 6 biological replicates). NC, negative control; KD, knockdown. Data are presented as mean ± SEM. ∗∗∗p < 0.005, n.s. not significant. (E) mRNA expression in *Gpr162* KD INS-1 832/13 cells (n = 6 biological replicates). NC, negative control; KD, knockdown. Data are presented as mean ± SEM. ∗∗∗p < 0.005. ∗ above column indicate comparisons with NC for the particular gene indicated. (F) *GPR162* KD in human islets (n = 7 donors). NC, negative control; KD, knockdown. Data are presented as mean ± SEM. ∗, p < 0.05, ∗∗p < 0.01. Data were analyzed using one-way ANOVA with Dunnett’s test (Figures 2B and 2C, 2D, 2E, and 2F).

Having established that CART binds to GPR162, we next tested whether CART-induced biological effects could be reduced after KD of *Gpr162* mRNA. First, we employed whole-cell capacitance measurements in INS-1 832/13 cells treated with CARTp55-102 for 1 h. In agreement with our previous findings,^8^ control cells responded to 10^−7^M CARTp55-102 with an approximately 1.3-fold increase in capacitance (Figures 2A and 2B). Notably, when *Gpr162* mRNA was knocked down, the CART-induced increase in capacitance was abolished (Figures 2A and 2B).

### Knockdown of *Gpr162* abolishes the effect of CART on insulin secretion

*Gpr162* KD per se resulted in increased insulin secretion at 16.7 mM glucose (with or without IBMX) in INS-1 832/13 cells (Figure 2C; replicated with another siRNA in Figure S1F). Furthermore, the previously established^8^ insulinotropic effect of CARTp55-102 on glucose and IBMX-stimulated insulin secretion was abolished in *Gpr162* KD cells (Figure 2D). *Gpr162* KD per se was also found to increase *Ins1*, *Ins2*, and *Cartpt* mRNA in INS-1 832/13 cells (Figure 2E; replicated with additional siRNA Figure S1E). The effect on insulin transcription was replicated in human islets treated with siRNA against *GPR162* (Figure 2F).

### CART and GPR162 knockdown affects density of actin filaments in the beta cell

**Figure 3:**
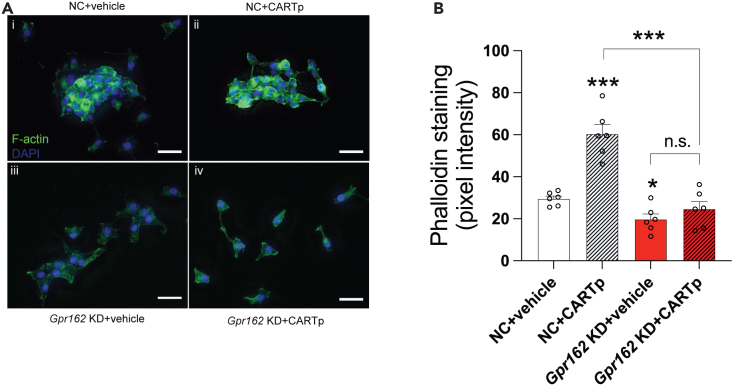
CARTp affects the cytoskeleton in INS-1 832/13 cells in a GPR162-dependent manner (A) Actin staining in INS-1 832/13 incubated for 24 h with CARTp (10^−7^M) (green; nuclei counterstained with DAPI) (panel i and ii). (panel iii and iv) *Gpr162* KD INS-1 cells incubated for 24 h with CARTp (10^−7^M) (green; nuclei counterstained with DAPI). (B) Quantification of (A). Scale bars 20 μm n = 6 biological replicates. Data are presented as mean ± SEM. ∗ above column indicates comparison with the NC + vehicle group, ∗ in brackets above two columns indicate the comparison made. NC, negative control; KD, knockdown. Data were analyzed using one-way ANOVA with Dunnett’s test (Figure 3B).

As *Cartpt* KD affected genes related to regulation of cytoskeletal processes (Tables S1A, S1B, and S2), we investigated a potential role for CART in regulating actin filament arrangement by culturing INS-1 832/13 cells for 24 h in the presence of 10^−7^M CARTp55-102. Control cells responded to the CARTp-incubation with an approximately 2-fold increase in actin staining, while CARTp was without effect in *Gpr162* KD cells (Figures 3A and 3B). Thus, our data show that CART binds to GPR162 and that beta cell effects of CART are reduced or abolished after *Gpr162* KD, suggesting a role for GPR162 in CARTp-mediated signaling in beta cells.

To shed light on the function of *Gpr162* in beta cells, we performed RNA sequencing of *Gpr16*2 KD INS-1 832/13 cells and cells treated with scrambled siRNA as controls. In total, 4929 genes were affected by *Gpr162* KD; 2461 genes were upregulated and 2468 were downregulated (Tables S3A and S3B). Corroborating our qPCR data, *Cartpt* was markedly upregulated in *Gpr162* KD INS-1 832/13 cells (padj = 3.12∗10^−5^, 2.25-fold upregulated). In keeping with our hypothesis that GPR162 is a receptor for CART (i.e., a peptide known to affect insulin secretion), the GO term insulin secretion (GO:0050796) was enriched in *Gpr162* KD cells (3.1-fold enriched, padj = 0.0106). GO term analysis also revealed processes related to cytoskeletal architecture (e.g., *regulation of actin cytoskeleton organization* GO:0032956, *regulation of actin filament-based process* GO:0032970, *regulation of cytoskeleton organization* GO: 0051493, and *actin cytoskeleton organization* GO:0030036) to be enriched by *Gpr162* KD. These GO terms mirror the *Cartpt* KD RNA-seq data and support our observations of CART-induced actin rearrangement. In particular the GO term *cytoskeletal protein binding* (GO:0008092) contained a number of important cytoskeletal genes (e.g., *Afap1*, *Ckap5*, *Gsn*, *Rab3a*, *Rab3b*, *Rab11b*, and *Arpc4*) (Table S6).

**Figure 4:**
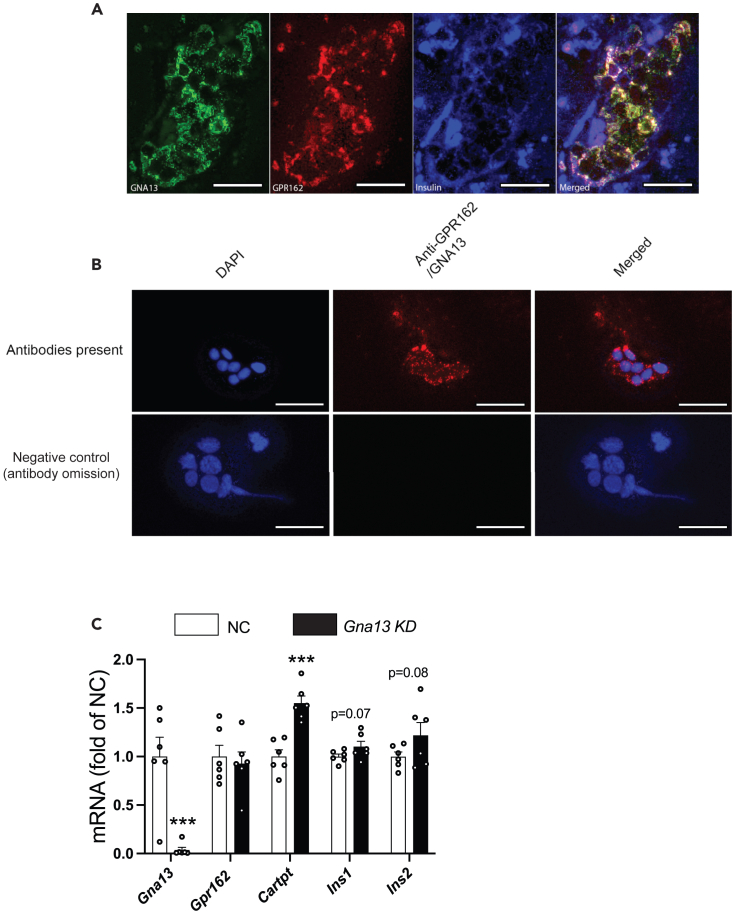
GPR162 interacts with GNA13 and knockdown of *Gna13* affects *Cartpt* mRNA in INS-1 832/13 cells (A) Triple immunostaining for GNA13, GPR162, and insulin in human pancreatic sections illustrating co-localization of GNA13 and GPR162 in beta cells (n = 4 samples). Scale bar is 50 μm. (B) Proximity ligand assay for GNA13 and GPR162 in INS-1 832/13 cells (n = 8 biological samples). Scale bar is 50 μm. (C) *Gna13* KD in INS-1 832/13 cells (n = 6 biological replicates). NC, negative control; KD, knockdown. Data are presented as mean ± SEM. ∗∗∗, p < 0.005. Data were analyzed using one-way ANOVA with Dunnett’s test (Figure 4C).

In an attempt to identify signaling molecules downstream of GPR162, we searched for *Cartpt* KD- and *Gpr162* KD-affected mediators of GPCR signaling in both the RNA-seq dataset. Of interest, G protein alpha 13 (*Gna13*) was found to be upregulated both by *Gpr162* KD (padj<0.05; 1.2-fold upregulated) and by *Cartpt* KD (padj<0.005; 1.68-fold upregulated). Notably, GNA13 was found to be co-localized with GPR162 in human beta cells (Figure 4A) and PLA in INS-1 832/13 cells confirmed an interaction between GNA13 and GPR162 (Figure 4B). *Gna13* KD caused 1.5-fold increased *Cartpt* mRNA expression and trends toward increased *Ins1* and *Ins2* mRNA expression (p = 0.07 and p = 0.08, respectively; Figure 4C).

## Discussion

We have previously shown that CART increases insulin secretion.^8^ However, the identity of the CART receptor mediating this effect in beta cells is unknown to date.^15^ Here, we provide evidence for GPR162 as a CART receptor in beta cells. We also demonstrate that the insulinotropic effect of CART is GPR162 dependent.

GPR162 was identified as the most CART-affected orphan receptor in INS-1 832/13 cells. Perhaps counterintuitive, *Cartpt* KD caused reduced expression of GPR162. On the other hand, *Gpr162* KD triggered a massive increase in *Cartpt* mRNA expression. By showing that CART binds to GPR162 and that biological effects of CART are reduced or ablated after *Gpr162* KD, we propose that GPR162 acts as a CART receptor in beta cells. We also established expression of GPR162 in a majority of human and mouse beta cells. Moderate GPR162 immunoreactivity was evident across the cytoplasm and more intense immunoreactivity was seen in cellular structures that remain to be deciphered. GPR162 is an orphan receptor belonging to the rhodopsin (class A) family of G protein-coupled receptors,^16^ previously shown to be expressed in the brain^17^ as well as in the heart and kidney.^18^ It is highly conserved between mammalian species, and human GRP162 has 95% amino acid identity with rodent orthologs.^19^ The identification of the CART receptor is long in the making. More than 25 years after the discovery of CART in 1995,^1^ no specific CART receptor has been put forward. A few clues as to the properties of this elusive receptor have been put forward over the years. Lakatos et al. presented evidence that exogenous CART was capable of activating AtT20 (a pituitary cell line)^20^ and studies using pertussis toxin suggested that the signaling of the CART receptor occurs through G_i/o_ proteins.^20^^,^^21^ G_i/o_-dependent signaling in beta cells was later questioned as CART was shown to increase cAMP in INS-1 832/13 cells.^22^^,^^23^

In the brain, it was recently suggested through a series of experiments that GPR160, another member of the class A family of GPCRs located on a different chromosome than GPR162, is a CART receptor in the brain.^11^^,^^12^ Information on GPR160 is scarce, but it has been reported to be a potential marker for prostate cancer,^24^ where it is involved in apoptosis and cell-cycle arrest.^25^ From an insulin perspective, it should be noted that it has previously been reported that GPR160 has no effect on C-peptide-induced cFOS expression in KATOIII cells.^26^ Recently, the notion of GPR160 being the CART receptor in the brain was put into question by Freitas-Lima et al. who investigated the ability of radiolabeled CART to bind in cell lines (THP1 cells and GPR160-transfected U2OS and U-251 MG cells) with high expression of GPR160.^13^ Also, these authors failed to detect *Gpr160* mRNA expression or GPR160 immunoreactivity in the PC12 cell line. The data presented by Freitas-Lima et al. are in line with our inability to detect GPR160 immunoreactivity in human islets or an interaction between CART and GPR160 in INS-1 832/13 cells.^13^ Sanchez-Navarro et al. recently reported that AAV-mediated knockdown of *Gpr160* in the dorsal vagal complex in the rat brain partially attenuates the anorexigenic effects of CART on meal microstructure.^27^ While we do not oppose the possibility that GPR160 is a CART receptor in the brain, further studies are needed to completely resolve the issue of the central CART receptor. In the present study, we propose that GPR162 serves as a CART receptor in the beta cell. This is based on the presence of GPR162 immunoreactivity in islet cells (in both rodent and human pancreas), an interaction between CART and GPR162, and reduced binding after *Gpr162* KD. The phenomenon of hormones having multiple, and sometimes even tissue-specific, receptors is not unusual; the plethora of 5-HT receptors being one example^28^ and the family of somatostatin receptors being another.^29^ Further studies are needed to understand whether GPR162 is also involved in mediating CART effects in the brain. It has been shown that GPR162 is widely expressed in, among other neurons, GABAergic neurons in the mouse hippocampus.^17^ Extensive expression has also been reported in other brain areas, such as the hypothalamus and amygdala, which regulate energy homeostasis and hedonic feeding.^17^ Of note, the hypothalamus is a brain region with significant expression of CART.^30^ Furthermore, sequence structure-based phylogeny has suggested GPR162 to be a receptor for adrenaline and noradrenaline.^31^ However, to our knowledge, this has not yet been functionally proven.

Interestingly, one report presents evidence in line with GPR162 being involved in regulation of glucose metabolism; Caruso et al. showed that *GPR162* genetic variants are associated with impairments in glucose homeostasis.^32^ Genotyping of four single-nucleotide polymorphisms of GPR162 in obese and normal-weight subjects of different ages revealed that variant rs2071081 in intron 4 is associated with impaired insulin levels and HOMA-IR in children.^32^

It has also been reported that intracerebroventricular injections of a *Gpr162* antisense oligodeoxynucleotide for 7 days results in decreased food intake in rats.^32^ Furthermore, it has been shown that diabetes affects the expression of *Gpr162* mRNA expression^18^ and that streptozotocin-treated rats displayed reduced *Gpr162* mRNA expression in the right ventricle of the heart and in the kidney, while the expression was increased in the brain.^18^ Also, GPR162 has been shown to increase the radiation-induced DNA damage response and is involved in the activation of the type I interferon system in carcinoma cell lines.^33^

Our observation that CART affects cytoskeletal rearrangement is novel. Without doubt, the actin network is of great importance for properly functioning insulin secretion.^34^^,^^35^^,^^36^^,^^37^ In the present study, a large number of genes involved in cytoskeletal rearrangement were affected by both *Cartpt* KD and *Gpr162* KD. We have previously demonstrated that CART causes increased synchronization of Ca^2+^ oscillations between beta cells in different parts within the same islet.^8^ Furthermore, *Cartpt* KD also affects genes crucial for regulation of exocytosis,^14^ a process indeed dependent on actin architecture. The observed effect of CARTp on actin architecture in INS-1 832/13 cells was lost in *Gpr162* KD cells, suggesting that CARTp exerts its cytoskeleton-modulating effects through Gpr162.

Along with de-orphanizing GPR162, our data also suggest that GPR162 signals downstream via GNA13. This is based on an observed GPR162-GNA13 interaction in INS-1 cells as well as co-localization of GPR162 and GNA13 in rodent and human beta cells. In addition, knockdown of *Gpr162* mRNA or *Gna13* mRNA affected CARTp-induced effects.^22^ GNA13 is reportedly more expressed in the liver than in other insulin target tissues.^38^ Mice with a liver-specific deletion of *Gna13* have been reported to display impaired glucose tolerance and noticeable insulin resistance when fed a high-fat diet,^39^ suggesting the involvement of GNA13 in glucose regulatory processes.

We also identified an interaction between CART and GPR162 using three different methods. The effect of *Gpr162* KD on this interaction ranged from 25% using radiolabeled CART to 90% using PLA. The observed difference is likely related to different sensitivity of the approaches. On the other hand, *Gpr162* KD caused a near-complete abolishment of the CART-induced effect on exocytosis, insulin secretion, and actin rearrangement. The PLA data is in accordance with the observed lack of biological effect, but we have no ready explanation for why we only observed a moderate reduction in CART-GPR162 interaction using radiolabeled CART. However, it is not inconceivable that it is related to the timing of siRNA treatments and/or incubation time during the binding assay.

In summary, we provide evidence for GPR162 as a CART receptor in beta cells. This is based on the expression of GPR162 in beta cells, interaction between CART and GPR162 using several approaches, and reduced or ablated effect of CART after *Gpr162* KD. In light of insulinotropic and glucagonostatic actions of CART, the identification of GPR162 as a beta cell CART receptor may open up avenues for novel strategies for the treatment of T2D.

### Limitations of the study

It should be mentioned that the present study has a few limitations. Firstly, the majority of experiments were performed in a cell line (INS-1 832/13 cells). Secondly, RNA sequencing does not reveal regulation on the protein level, and as such caution should be exercised when interpreting and extrapolating results obtained on the mRNA level to protein level. However, in this case, extrapolation from mRNA to protein seems to hold true, as mRNA levels and protein levels change in the same direction after knockdown. While the effects of exogenous CARTp were clearly reduced or abolished after *Gpr162* KD, *Gpr162* KD in itself caused increased insulin secretion. Thus, the potential existence and influence of other contributors and mediators of CART signaling in the beta cell cannot be ruled out.

## STAR★Methods

### Key resources table

**Table:** 

REAGENT or RESOURCE	SOURCE	IDENTIFIER
**Antibodies**		

Rabbit anti-glucagon	EuroDiagnostika, Malmö, Sweden	Cat#M8707
Rabbit anti-GNA13	Invitrogen, Waltham, MA	Cat#PA5-100664
Rabbit anti-GPR160	Invitrogen, Waltham, MA	Cat#PA5-33650
Rabbit anti-GPR162	Invitrogen, Waltham, MA	Cat#PA5-33655
Rabbit anti-GPR162	Santa Cruz, Dallas, TX	Cat#sc-138323
Rabbit anti-GPR162	Origene, Rockville, MD	Cat#TA331838
Guinea pig anti-insulin	EuroDiagnostika, Malmö, Sweden	Cat#M9003
Goat anti-CART	Santa Cruz, Dallas, TX	Cat#sc-18068
Rabbit anti-CART	Kind gift from Jes T Clausen, Novo Nordisk, Copenhagen, Denmark	2059A
Rabbit anti-GIPr	kind gift from professor Timothy Kieffer, Univ. of British Columbia, Vancouver, Canada	RbαGIPr551#4

**Biological samples**		

Human pancreas embedded in paraffin	Human Tissue Lab, Lund University	https://www.ludc.lu.se/human-tissue-lab
Isolated human islets (male donors)	Human Tissue Lab, Lund University	https://www.ludc.lu.se/human-tissue-lab

**Chemicals, peptides, and recombinant proteins**		

Human CARTp55-102	Phoenix Pharmaceuticals, Burlingame, CA	Cat#003-60
Rat, mouse CARTp55-102	Phoenix Pharmaceuticals, Burlingame, CA	Cat#003-62

**Critical commercial assays**		

NucleoSpin RNA Extraction kit	Macherey Nagel, Dueren, Germany	Cat#740955.250
NucleoSpin XS RNA Extraction kit	Macherey Nagel, Dueren, Germany	Cat#740902.250
Rat Insulin ELISA	Mercodia, Uppsala, Sweden	Cat#10-1250-01
Mouse Insulin ELISA	Mercodia, Upsala, Sweden	Cat#10-1247-01
SuperSignal West Femto	BioRad, Hercules, CA	Cat#34095

**Deposited data**		

*RNA sequencing Cartpt* KD INS-1 raw data	This paper	NCBI GEO: GSE245796
RNA sequencing *Gpr162* KD INS-1 raw data	This paper	NCBI GEO: GSE245691

**Experimental models: Cell lines**		

Rat: INS-1 832/13 (insulinoma of male origin)	kind gift from prof. Hindrik Mulder, Lund University, Sweden	N/A
TaqMan for rat *Gpr162*	Life Technologies, Carlsbad, CA	Cat#Rn01459205
TaqMan for rat *Gna13*	Life Technologies, Carlsbad, CA	Cat#Rn01461471
TaqMan for rat *Ins1*	Life Technologies, Carlsbad, CA	Cat#Rn02121433
TaqMan for rat *Ins2*	Life Technologies, Carlsbad, CA	Cat#Rn01774648
TaqMan for rat *Hprt1*	Life Technologies, Carlsbad, CA	Cat#Rn01527840
TaqMan for rat *Tbp*	Life Technologies, Carlsbad, CA	Cat#Rn01455646
TaqMan for human *GPR162*	Life Technologies, Carlsbad, CA	Cat#Hs00973214
TaqMan for human *INS*	Life Technologies, Carlsbad, CA	Cat#Hs00355773
TaqMan for human *HPRT1*	Applied BioSystems, Waltham, MA	Cat#4326321E-1010014
TaqMan for human *TBP*	Life Technologies, Carlsbad, CA	Cat#Hs004427620
siRNA for rat *Cartpt*	Life Technologies, Carlsbad, CA	Cat#s130729
siRNA for rat *Cartpt*	Life Technologies, Carlsbad, CA	Cat#s130728
siRNA for rat *Gpr162*	Life Technologies, Carlsbad, CA	Cat#s169118
siRNA for rat *Gpr162*	Life Technologies, Carlsbad, CA	Cat#s169117
siRNA for rat *Gna13*	Life Technologies, Carlsbad, CA	Cat#s155399

**Software and algorithms**		

GraphPad Prism 8 and 9	GraphPad software, Boston, MA	https://graphpad.com
ImageJ	National Institutes of Health, Bethesda, MA and Laboratory for Optical and Computational Instrumentation, University of Wisconsin, Madison, WI	https://imagej.nih.gov/ij/index.html
CellSens Elements	Olympus, Tokyo, Japan	N/A

### Resource availability

#### Lead contact

Further information and requests for resources and reagents should be directed to and will be fulfilled by the Lead Contact, Professor Nils Wierup (nils.wierup@med.lu.se).

#### Materials availability

This study did not generate new unique reagents.

#### Data and code availability

• All data reported in the paper will be shared by the lead contact upon request.
• Any additional information required to reanalyze the data reported in the paper is available from the lead contact upon request. All raw and processed RNA-seq data is deposited in the GEO database and is publicly available as of the date of publication. Accession numbers are listed in the key resources table.
• This paper does not report original code.

### Experimental model and study participant details

Mouse islets were isolated under an ethical permit approved by the local Animal Welfare Committee (Permit no. 6508-21). Human islets were provided by the Nordic Network for Clinical Islet Transplantation and the EXODIAB Human Tissue Lab. Lund University Diabetes Center (LUDC) holds a permit for the use of these islets (Permit no. 2019-00357).

### Method details

#### RNAsequencing

RNA quality was assessed using the 2200 TapeStation (Agilent Technologies, Santa Clara, CA). One microgram of total RNA of sufficient quality RNA (RIN>8) was used for sample preparation for sequencing with a TrueSeq RNA sample preparation kit (Illumina Inc., San Diego, CA). Transcriptome sequencing was performed on the Illumina HiSeq 2000 platform using a paired-end 101 bp protocol (Illumina). A library was constructed using Illumina TrueSeq sample preparation kit, paired end (Illumina). Amplification was performed for 101 cycles. Six libraries were multiplexed on each line of the flow cell. The average number of paired-end reads for each sample obtained was 32 million reads. Reads were mapped by STAR (v2.4.1) to the rat reference, Rattus_norvegicus v. 6.0.83, downloaded from ENSEMBL).

#### *In vitro* cell culture

INS-1 832/13 cells (a rat insulinoma cell line of male origin) were cultured in RPMI 1640 medium (Sigma Aldrich, St. Louis, MO) containing 2 g/L D-glucose, supplemented with 10% FBS, 10 mM HEPES, 1 mM sodium pyruvate and 50 μM β-mercaptoethanol (Sigma Aldrich) in a humidified atmosphere (37°C, 5% CO_2_). For investigations of actin architecture, INS-1 832/13 cells were seeded in 8-well chamber slides (Sarstedt) and transfected as described below before being fixed, permeabilized and stained with phalloidin (Phalloidin-iFluor488, Abcam, Cambridge, UK) for 60 min. Nuclei were counter-stained with DAPI and F-actin was visualized using Phalloidin-iFluor488 (Abcam). Phalloidin staining was performed for 60 min at a dilution of 1:1000 (in PBS +0.25% Triton X-100). Phalloidin intensity was quantified using the ImageJ software.

#### Tissue handling

Human or mouse pancreata were either fixed in 4% paraformaldehyde for 24 h (then placed in 70% ethanol) or fixed in Stefanini's solution (2% paraformaldehyde and 0.2% picric acid in 0.1 M PBS, pH 7.2) for 24 h (then rinsed thoroughly in Tyrode's solution containing 10% sucrose). Mice were lightly anaesthesized with Isofluran (Abbott, Berkshire, UK) and then cervically dislocated. Pancreas was dissected out and placed in either Stefanini's or 4% paraformaldehyde. Sections were cut to a thickness of 6 μm. All procedures were approved by the Animal Ethics Committee in Lund and Malmö.

#### Immunohistochemistry

The following primary antibodies were used: guinea-pig anti-glucagon code M8707, dilution 1:2500 (MiLab, Sweden), rabbit anti-GNA13, code PA5-1000664, dilution 1:500 (Invitrogen, Waltham, MA); rabbit anti-GPR160, code PA5-33650, dilution 1:500 (Invitrogen); rabbit anti-GPR162, code PA5-33655, dilution 1:500 (Thermo Scientific); guinea pig anti-insulin, code M9003, dilution 1:2500 (EuroDiagnostika, Malmö, Sweden). The specificity of the GPR162 antibody was investigated with an additional rabbit anti-GPR162 antibody (code SAB2900470, dilution 1:500) from Sigma Aldrich (St Louis, MI) and a goat anti-GPR162 antibody (code sc-138323, dilution 1:500) from Santa Cruz (Dallas, TX) (see Figure S1). To further verify specificity, pre-absorption with a blocking peptide (NBP2-33740PEP) from Novus Biological was performed. Antibodies were diluted in PBS containing 0.25% BSA and 0.25% Triton X-100.

Pancreatic sections were incubated with primary antibodies overnight at 4°C, followed by rinsing in PBS with 0.25% Triton X-100 for 2 X 10 min. Thereafter, secondary antibodies (donkey anti-rabbit Cy2 for GNA13, GPR160 and GPR162, donkey anti-goat Texas Red for GPR162 and donkey anti-guinea pig Alexa Fluor 594 for insulin and glucagon) were applied for 1 h at room temperature. Sections were again rinsed in PBS with 0.25% Triton X-100 and mounted in PBS:glycerol (1:1). Secondary antibodies were purchased from Jackson Immunoresearch (West Grove, PA) (Cy2) and Life Technologies (Alexa Fluor 594). Cy2 and Alexa Fluor 594 were used at a dilution of 1:400.

#### Proximity ligation assay (PLA)

INS-1 832/13 cells were seeded in 8-well tissue culture chambers (Sarstedt, Nümbrecht, Germany). 10^−7^M rat CARTp55-102 (Phoenix Pharmaceuticals, Burlingame, CA) was added to fresh media and cells were incubated for 5 min in a humidified atmosphere (37°C, 5% CO_2_). Cells were rinsed quickly in ice-cold 1XPBS and then washed twice with ice-cold PBS. Cells were then fixed for 20 min in 4% paraformaldehyde, washed 2 × 10 min in 1XPBS and permeabilized for 15 min in PBS +0.25% Triton X-100. Proximity ligation assay (PLA) (DuoLink *In situ* Red Starter Kit Goat/Rabbit, Sigma Aldrich) was performed according to the instructions provided by the manufacturers. The following antibody combinations were used for the PLA: goat anti-GPR162 (sc-138323, dilution 1:500, Santa Cruz) with rabbit anti-CART (code 2059A, dilution 1:5000, kindly provided by Dr. Jes T. Clausen, Novo Nordisk, Måløv, Denmark), and goat anti-CART (code sc-18068, dilution 1:400, Santa Cruz) with rabbit anti-GPR162 (code PA5-33655, dilution 1:500, Thermo Scientific). Anti-rabbit GPR160 (code PA5-33650) was combined with anti-goat CART (code sc-18068 from Santa Cruz). Anti-rabbit GNA13 (PA5-100644) was combined with anti-goat GPR162 (sc-138323). Negative controls were omission of both antibodies and the combination of the CART antibody with an antibody toward a receptor with a known ligand. In our case we combined goat anti-CART (code sc-18068, dilution 1:400, Santa Cruz) with rabbit anti-GIP receptor (code RbαGIPr551#4, dilution 1:400, kindly provided by Prof. Timothy Kieffer, University of British Columbia, Vancouver, Canada).

#### Radiolabeling of CARTp55-102

^125^I was incorporated into the meta-position of tyrosine side chains of CARTp55-102 using oxidative iodination. 20 μL of a 300 mM phosphate buffer (pH 7.4) was used to resuspend 1 nmol of the CARTp55-102 peptide, after which 0.4 mCi ^125^I (NEZ033A, PerkinElmer, Waltham, MA) was added. A volume of 30 μL 3 mg/mL Chloramine-T in phosphate buffer was added gradually while stirring over 6 min, and the reaction was quenched by adding 400 μL H_2_O with 0.1% trifluoroacetic acid (TFA). The radiolabeled peptide was purified by reversed-phase HPLC on a C18 column and an acetonitrile column. Elution fractions of 1 mL were collected and it was determined where the radiolabeled peptide eluted by measuring γ radiation intensity on a PerkinElmer 2470 Automatic Gamma Counter (PerkinElmer).

#### Radioactive binding

INS-1 cells were seeded to 24-well plates and transfected with *Gpr162* siRNA (or control siRNA). Two days after transfection cells were assayed for competition binding for 3 h at 4°C using 10–15 p.m. ^125^I CARTp55-102 or unlabeled CARTp55-102 in 0.4 mL of 50 mM HEPES buffer (pH7.4 supplemented with 1 mM CaCl_2_, 5 mM MgCl_2_ and 0.5% (w/v) bovine serum albumin). After incubation cells were washed two times in binding buffer supplemented with 0.5 M NaCl at 4°C. Non-specific binding was determined as the binding in the presence of 0.1μM unlabeled CARTp55-102.

#### Coimmunoprecipitation

INS-1 832/13 cell lysates were incubated with 10^−7^M CARTp55-102 for 30 min at 4°C and then cross-linked with 1 mM BS^3^ for 30 min at room temperature, then precipitated using biotin-labelled CART antibody (code H003-62, dilution 1:5000, Phoenix Pharmaceuticals) bound to streptavidin-coated magnetic Dynabeads (Thermo Fisher). Cells lysates were then washed five times in ice-cold PBS and membrane proteins were extracted using MemPER Plus (Thermo Scientific). Protein concentration was then measured using BCA assay (BioRad, Hercules, CA) and 20 μg loaded on a 10% Mini-PROTEAN TGX Stain-Free gel (BioRad). After electrophoresis (200 V for 40 min) and transfer, the PVDF membrane was blocked in TBS-T with 5% fat free dry milk. Primary antibody (anti-rabbit GPR162, code TA331838, 1:100 dilution, Origene, Rockville, MD) was dissolved in 1XTBS-T with 5% BSA (overnight at 4°C). Secondary antibody (anti-rabbit IgG; 1:2500 dilution) was diluted in 1XTBS-T with 5% dry milk. All wash steps were for 10 min in 1XTBS-T.

#### Electrophysiology

Whole-cell capacitance measurement was performed in INS-1 832/13 cells using HEKA EPC10 patch-clamp amplifiers with the software Suite Pulse + X-Chart Extension (version 8.6 or later; HEKA, Lambrecht-Pfalz, Germany) as previously described.^40^ Briefly, INS-1 832/13 cells were seeded in Nunc plastic petri dishes one day prior to the measurement. The intracellular pipette solution contained 125 mM Cs-glutamate, 10 mM CsCl, 10 mM NaCl, 1 mM MgCl_2_, 5 mM HEPES, 3 mM Mg-ATP, 0.1 mM cAMP and 0.05 mM EGTA (pH 7.2 with CsOH). The perfusing extracellular solution was composed of 118 mM NaCl, 20 mM tetraethylammonium chloride, 5.6 mM KCl, 2.6 mM CaCl_2_, 1.2 mM MgCl_2_, 5 mM HEPES and 5 mM glucose (pH 7.4), and the temperature was maintained at 32°C. The whole-cell configuration was used in voltage-clamp mode and pipettes had an average resistance of ≈5.5 MΩ.

#### Imaging and morphometry

Immunofluorescence was examined in an epifluorescence microscope (Olympus BX60, Olympus, Tokyo, Japan) with a digital camera (Olympus DP74, Olympus).

#### Western blotting experiments

INS-1 832/13 cells were seeded in 6-well plates and transfected with *Cartpt* siRNA. 72 h after transfection cells media was removed and cells washed in 1 X PBS. 200 μL cOmplete lysis buffer (Roche, Basel, Switzerland) was added and cells were stored at −20°C until analysis.

#### Western blotting

Twenty micrograms of cell lysates were separated on a 10% Mini-PROTEAN TGX Stain-Free gel (BioRad). After transfer using the Trans-Blot Turbo RTA Transfer kit (BioRad) and blocking in TBS-T with 5% (w/v) non-fat dry milk, the PVDF membrane was incubated overnight at 4°C with anti-rabbit GPR162 antibody (code TA331838, 1:100 dilution, Origene) diluted in TBS-T with 5% (w/v) BSA. Membranes were then washed 3 × 10 min in TBS-T at room temperature, after which they were incubated for 60 min at room temperature with secondary antibodies diluted in TBS-T with 5% dry milk (goat anti-rabbit IgG, 1:2500, Prod#32460, Thermo Scientific). Membranes were again washed 3 × 10 min in TBS-T and then developed using SuperSignal West Femto Maximum Sensitivity Substrate from Thermo Scientific. Images were obtained using the ChemiDoc MP Imaging system (BioRad).

#### Glucose-stimulated insulin secretion (GSIS) in INS-1 832/13

GSIS was measured 72 h after *Cartpt* KD. INS-1 832/13 cells were washed twice and incubated for 2 h in 2.8 mM glucose HEPES-buffered saline solution (HBSS): 114 mM NaCl, 4.7 mM KCl, 1.2 mM KH_2_PO_4_, 1.16 mM MgSO_4_, 20 mM HEPES, 2.5 mM CaCl_2_, 25.5 mM NaHCO_3_, 0.2% BSA, pH 7.2, followed by 1-h stimulation in the same buffer containing 2.8 mM, 16.7 mM glucose, 16.7 mM glucose and 10 μM IBMX. In a separate experiment, control and *Gpr162* KD cells were incubated with 10^−7^M CARTp55-102 for 24 h before GSIS. Insulin concentrations in supernatant were determined using ELISA (Mercodia, Uppsala, Sweden) and were normalized to total protein content of each well (determined by Bio-Rad protein assay, BioRad).

#### Insulin ELISA

Insulin from INS-1 832/13 cells and mouse islets were analyzed using ELISA (Mercodia).

#### siRNA-mediated gene knock down and real-time qPCR in INS-1 832/13

Gene knockdown in INS-1 832/13 cells was performed using Lipofectamin RNAiMAX (#13778150, Life Technologies) and 60 nM siRNA targeting rat *Cartpt* (s130729, Silencer Select Pre-designed siRNA), *Gpr162* (s169118, Silencer Select Pre-designed siRNA) or *Gna13* (s155399, Silencer Select Predesigned). The sequences for scrambled siRNA were sense: 5′-GAGACCCUAUCCGUGAUUAtt-3′ and antisense: 5′-UAAUCACGGAUAGGGUCUCtt-3’ (Silencer Select customer designed siRNA). The transfection complexes were prepared according to the manufacture’s protocol. Total RNA was isolated 48 h after transfection using the NucleoSpin kit from Nagel-Macherey (Duren, Germany) and 1 μg of RNA was reverse-transcribed to cDNA using RevertAid First Strand cDNA synthesis kit (#K1622, Thermo Scientific). Quantitative RT-PCR was with 25 ng cDNA and TaqMan gene expression assays (Life Technologies; Rn01645174 for *Cartpt*, Rn01459205 for *Gpr162*, Rn01461471 for *Gna13*, Rn02121433 for *Ins1*, Rn01774648 for *Ins2*). All samples were analyzed with two endogenous controls (Rn01527840 for *Hprt1* and Rn01455646 for *Tbp*). The gene expression levels were determined using the 2^−ΔΔCt^ method. Key experiments were replicated using additional siRNAs (s130728 for *Cartpt* and s169117 for *Gpr162*; both Silencer Select Pre-designed siRNA from Life Technologies).

#### siRNA knockdown in human islets

Islets were picked clean and transferred to 35-mm dishes with fresh medium (CMRL1066 supplemented with 10 nM nicotinamide, 10 mM HEPES, 0.25 μg/mL fungizone, 50 μg/mL gentamicin, 2 mM L-glutamine, 10 μg/mL ciprofloxacin and 10% v/v heat-inactivated human serum). On the day of transfection islets (100 islets/dish) were transferred to dishes containing transfection media (CMRL1066 without supplementation). 60 nM of siRNA and Lipofectamine RNAiMAX was used. The following day, cells were transferred to fresh supplemented medium. RNA was extracted 96 h after transfection using the NucleoSpin XS kit (Nagel-Macherey). The siRNA for *GPR162* (s26015) was purchased from Life Technologies. The sequences for scrambled siRNA were sense: 5′-GAGACCCUAUCCGUGAUUAtt-3′ and antisense: 5′-UAAUCACGGAUAGGGUCUCtt-3’ (Silencer Select customer designed siRNA, Ambion, Life Technologies). TaqMans used were from Life Technologies and were: Hs00973214 for *GPR162*, Hs00355773 for *INS* and Hs004427620 for *TBP*. *HPRT1* (4326321E-1010014) was from Applied Biosystems. Gene expression levels were determined using the 2^−ΔΔCt^ method.

### Quantification and statistical analysis

Images were quantified using ImageJ and CellSens Elements (Olympus) software. One-way ANOVA with Dunnett's test or Student's t-test was used where appropriate. Data are presented as mean ± SEM. Differences were considered statistically significant if p < 0.05.

## Supplemental information

Document S1. Figures S1–S3

Table S1. Genes upregulated by *Cartpt* KD, related to Figure 1

Table S2. Genes downregulated by *Cartpt* KD, related to Figure 1

Table S3. GO terms affected by *Cartpt* KD, related to Figure 1

Table S4. Genes upregulated by *Gpr162* KD, related to Figure 1

Table S5. Genes downregulated by *Gpr162* KD, related to Figure 1

Table S6. GO terms affected by *Gpr162* KD, related to Figure 1
