# Supplementary material for: GPR162 is a beta cell CART receptor
Source: iScience. 2023 Nov 10;26(12):108416. doi: 10.1016/j.isci.2023.108416 (PMC10709007; doi:10.1016/j.isci.2023.108416)
Supplement: Document S1. Figures S1–S3 [file mmc1.pdf]

iScience, Volume *26*

Supplemental information

GPR162 is a beta cell CART receptor

Andreas Lindqvist, Mia Abels, Liliya Shcherbina, Mtakai Ngara, Dmytro Kryvokhyzha, Sabrina Chriett, Matteo Riva, Abul Fajul, Mohammad Barghouth, Cheng Luan, Lena Eliasson, Olav Larsen, Mette M. Rosenkilde, Enming Zhang, Erik Renström, and Nils Wierup

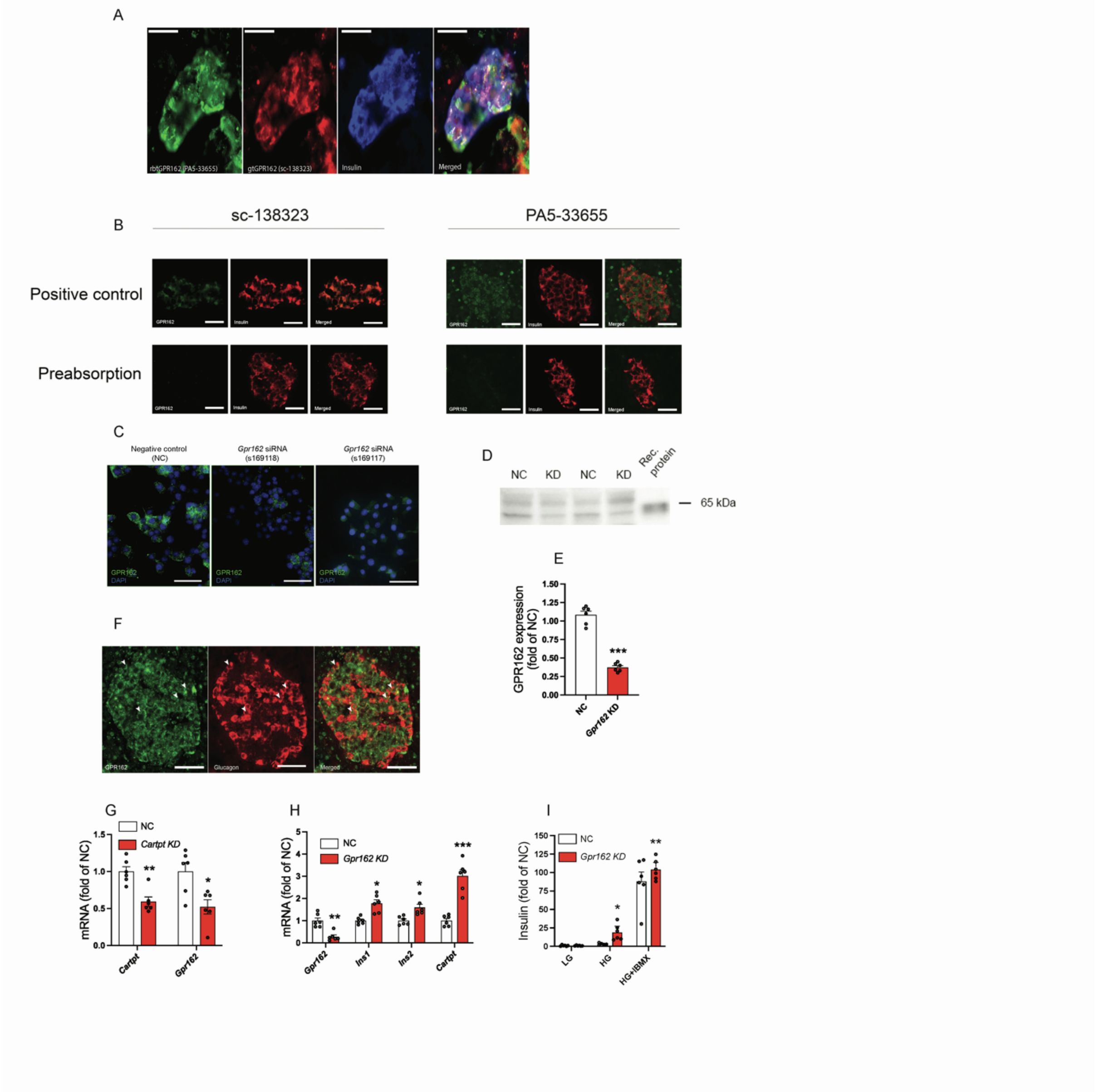

Figure S1. Replication of key findings of the manuscript using additional antibodies and siRNAs for GPR162. Related to Figure 1.

(A) Immunostaining showing the specificity of the GPR162 antibodies. The rabbit GPR162 antibody from ThermoFisher (green) showed complete overlap with the goat GPR162 antibody from Santa Cruz (red) in beta cells (insulin in blue) in human pancreatic sections (n=5 samples). Scale bar is 50 μm.

(B) Preabsorption of both GPR162 antibodies resulted in absence of immunoreaction, thus further illustrating their specificity (n=5 samples). Scale bar is 50 μm.

(C) *Gpr162* KD in INS-1 832/13 cells (n=6 biological replicates) using two siRNAs. NC, negative control. Scale bar is 50 μm.

(D) Representative GPR162 Western blot in *Gpr162* KD INS-1 832/13 cells (n=6 biological replicates. NC, negative control; KD, knock down. Rec. protein, recombinant GPR162 protein as control.

(E) quantification of (D). NC, negative control; KD, knock down. Data are presented as mean±SEM. ***p<0.005.

(F) GPR162 expression in alpha cells in human pancreatic sections (n=5 samples). Scale bar is 50 μm.
(G) Replication of the effect of *Cartpt* KD on *Gpr162* mRNA using another siRNA (n=6 biological replicates). NC, negative control; KD, knock down. Data are presented as mean±SEM. *p<0.05, **p<0.01.
(H) Replication of *Gpr162* knockdown on *Ins1*, *Ins2* and *Cartpt* mRNA was replicated using another siRNA (n=6 biological replicates). NC, negative control; KD, knock down. Data are presented as mean±SEM. *p<0.05, **p<0.01, ***p<0.005.
(I) Replication of glucose-stimulated insulin secretion in *Gpr162* KD INS-1 832/13 cells using another siRNA (n=6 biological replicates). NC, negative control; KD, knock down. Data are presented as mean±SEM. *p<0.05, **p<0.01.

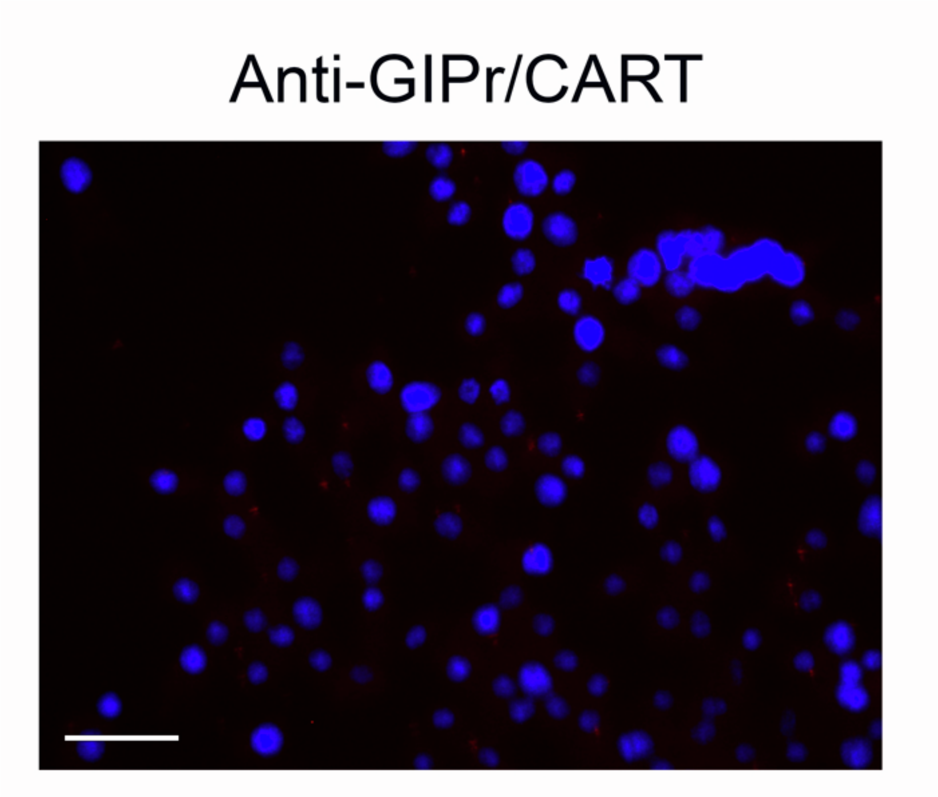

Figure S2. PLA for CART-GIPr. Related to Figure 1.
Proximity ligation assay for GIPr and CART (n=8 biological replicates). Scale bar is 20 µm.

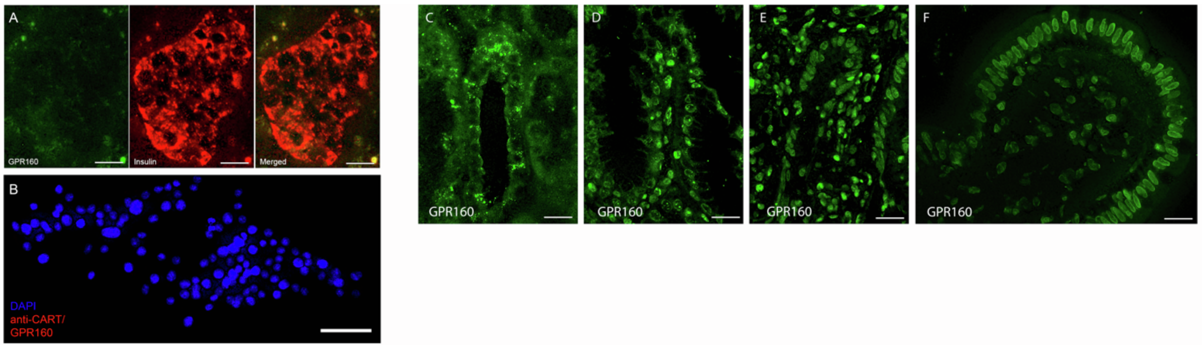

Figure S3. GPR160 expression is not detected in human beta cells using neither immunohistochemistry nor PLA. Related to Figure 1.

(A) Immunohistochemistry for GPR160 (green) in human pancreatic sections (insulin in red to visualize beta cells, n=5 samples). Scale bar is 50 µm.

(B) PLA in INS-1 832/13 cells incubated with 10^{-7}M CARTp55-102 for 15 minutes shows no interaction between GPR160 and CART (B, n=8 biological replicates). Scale bar is 20 µm.

(C) GPR160 expression in human kidney (n=4 samples). Scale bar is 50 µm

(D) GPR160 expression in human stomach (n=4 samples). Scale bar is 50 µm

(E) GPR160 expression in human ileum (n=4 samples). Scale bar is 50 µm

(F) GPR160 expression in human jejunum (n=4 samples). Scale bar is 50 µm.
